# Three New Species of the Genus *Ochroconis*

**DOI:** 10.1007/s11046-015-9910-5

**Published:** 2015-06-21

**Authors:** K. Samerpitak, A. H. G. Gerrits van den Ende, S. B. J. Menken, G. S. de Hoog

**Affiliations:** CBS-KNAW Fungal Biodiversity Centre, Uppsalalaan 8, 3584CT Utrecht, The Netherlands; Institute for Biodiversity and Ecosystem Dynamics, University of Amsterdam, Amsterdam, The Netherlands; Department of Microbiology, Faculty of Medicine, Khon Kaen University, Khon Kaen, Thailand; Research Center for Medical Mycology, Peking University Health Science Center, Beijing, China; Sun Yat-sen Memorial Hospital, Sun Yat-sen University, Guangzhou, China; Second Medical Military University, Shanghai, China; Basic Pathology Department, Federal University of Paraná State, Curitiba, Paraná Brazil; King Abdulaziz University, Jeddah, Saudi Arabia

**Keywords:** *Ochroconis*, Ant fungus, Opportunist, Phylogeny, Venturiales

## Abstract

*Ochroconis bacilliformis*, *O. phaeophora* and *O. robusta*, three novel species of the melanized genus *Ochroconis* (*Sympoventuriaceae*, *Venturiales*), are described, illustrated and distinguished phenotypically and molecularly from previously described species in the genus *Ochroconis*. Their potential significance for infection of cold-blooded vertebrates is discussed.

## Introduction

The genus *Ochroconis*, typified by *O. constricta*, was morphologically segregated from a genus with lobed conidia, *Scolecobasidium* by de Hoog & von Arx [1], for melanized fungi with sympodial conidiogenesis and septate, ellipsoidal conidia which were liberated rhexolytically. Machouart et al. [2] elucidated the general phylogenetic position of the genus by investigating highly conserved genes (nuSSU, nuLSU, mtSSU and *RPB2*) and found that *Ochroconis* and its relatives belonged to the order *Venturiales*, family *Sympoventuriaceae*. Samerpitak et al. [3] studied species diversity by analyzing more variable genes in addition to the partial ribosomal operon, i.e., the partial coding genes, actin (*ACT1*), β-tubulin (*BT2*) and translation elongation factor 1-α (*TEF1*) and recognized thirteen species in *Ochroconis*. The authors also introduced the genus *Verruconis* for a group of thermophilic species such as *Ochroconis gallopava*, an opportunistic neurotropic pathogen, and its sibling, *O. calidifluminalis* [4]. The taxonomic status of *Scolecobasidium* was considered to be doubtful because of ambiguity of the type material, *S. terreum* [3]. The strict morphological parameters to demarcate the genera were abandoned at the expense of a phylogenetic approach. Species with forked conidia similar to *S. terreum* were added to *Ochroconis* on phylogenetic grounds as members of *Sympoventuriaceae*. Some of the species that were morphologically classified in *Scolecobasidium* are currently not available for sequencing, and their classification remains unresolved.

Relatively large phylogenetic distances were noted among and within species of *Ochroconis* and *Verruconis* [3], which indicated the possible existence of additional, presently unrecognized taxa. Giraldo et al. [5] reported three new species, *O. icarus*, *O. ramosa* and *O. olivacea*, during an investigation of *Ochroconis* and *Verruconis* strains originating from clinical samples. The first two are sister species of *O. minima*, while the latter is close to but significantly different from *O. verrucosa*. Samerpitak et al. [6] proposed a further new species, *O. globalis*, which was phylogenetically close to but morphologically different from *O. tshawytschae*. Crous et al. [7] described a new species, *O. macozamiae*, as a sister species of *O. gamsii* which has similar morphology but different genotype.

This article presents a taxonomic study of three *Ochroconis* strains from various sources. Phenotypic and genotypic characters of the strains were evaluated to define species concepts and delimitations, and three novel *Ochroconis* species are proposed.

## Materials and Methods

### Phenotypic Studies

Three *Ochroconis* strains: CBS 112.97, CBS 206.96 and CBS 100442 (Table 1), were cultured on oatmeal (OA) and malt extract agars (MEA) and incubated at 24 °C for 21 days. Morphological observations were carried out as described by Samerpitak et al. [3, 6]. To investigate the optimal temperature for growth, all strains were grown on MEA incubated for 3 weeks at temperatures varying from 4 to 40 °C with 3 °C intervals. Colony diameters were measured after 3, 7, 11, 14, 18 and 21 days.

**Table 1:** Strains included in the study

Species	Strain	GenBank accession numbers		
		SSU	ITS	LSU
*Ochroconis* sp. 1	CBS 112.97	KP798639	KP798633	KP798636
*Ochroconis* sp. 2	CBS 206.96	KP798637	KP798631	KP798634
*Ochroconis* sp. 3	CBS 100442	KP798638	KP798632	KP798635
*O. anellii*	CBS 284.64 (T)	KF156070	FR832477	KF156138
*O. anomala*	CBS 131816 (T)	KF156065	HE575201	KF156137
*O. constricta*	CBS 202.27 (T)	KF156072	AB161063	KF156147
*O. cordanae*	CBS 475.80 (T)	KF156058	KF156022	KF156122
*O. crassihumicola*	CBS 120700	KJ867431	KJ867429	KJ867430
*O. gamsii*	CBS 239.78 (T)	KF156088	KF156019	KF156150
*O. globalis*	CBS 119644 (T)	KF961108	KF961086	KF961097
*O. humicola*	CBS 116655 (T)	KF156068	HQ667521	KF156124
*O. icarus*	CBS 536.69 (T)	KF156084	HQ667524	KF156132
*O. lascauxensis*	CBS 131815 (T)	KF156069	FR832474	KF156136
*O. longiphora*	CBS 435.76	KF156060	KF156038	KF156135
*O. macrozamiae*	CBS 102491	KF156092	KF156021	KF156152
*O. minima*	CBS 510.71 (T)	KF156087	HQ667522	KF156134
*O. musae*	CBS 729.95	KF156082	KF156029	KF156144
*O. olivacea*	CBS 137170 (T)	LM644548	LM644521	LM644564
*O. ramosa*	CBS 137173 (T)	LM644551	LM644524	LM644567
*O. sexualis*	CBS 131765 (T)	KF156089	KF156018	KF156118
*O. tshawytschae*	CBS 100438 (T)	KF156062	HQ667562	KF156126
*O. verrucosa*	CBS 383.81 (T)	KF156067	KF156015	KF156129
*Verruconis calidifluminalis*	CBS 125818 (T)	KF156046	AB385698	KF156108
*V. gallopava*	CBS 437.64 (T)	KF156053	HQ667553	KF156112
*V. verruculosa*	CBS 119775	KF156055	KF156014	KF156106
*Fusicladium sicilianum*	CBS 105.85 (T)	KP798640	FN549914	FN398150
*Scolecobasidium excentricum*	CBS 469.95 (T)	KF156096	HQ667543	KF156105
*Veronaeopsis simplex*	CBS 588.66 (T)	KF156095	KF156041	KF156103
*Sympoventuria capensis*	CBS 120136 (T)	KF156094	KF156039	KF156104
*Venturia inaequalis*	CBS 594.70 (T)	KF156093	KF156040	GU301879

*(T)* Type strain, *CBS* Centraalbureau voor Schimmelcultures, Utrecht, The Netherlands

### Phylogeny

Three *Ochroconis* strains: CBS 112.97, CBS 206.96 and CBS 100442, including twenty-seven type and reference strains of *Ochroconis*, *Verrconis* and neighboring genera (Table 1) were included in phylogenetic analyses. DNA extraction was performed [8–10], three markers, viz. nuSSU, nuLSU and ITS, were amplified by PCR using primers and conditions as shown in Table 2, and sequencing was performed by Big Dye Terminator Cycle Sequencing RR mix protocol (Applied Biosystems). BioNumerics v. 4.61 (Applied Maths, Sint-Martens-Latem, Belgium) was employed for first iterative alignments. Sequences of nuLSU, nuSSU and ITS were aligned with the web-based program Muscle (www.ebi.ac.uk/Tools/msa/muscle). Sequence alignments were adjusted using BioEdit v. 7.0.5.2. Guanine–cytosine content (G+C %) of ITS was calculated using BioEdit v. 7.0.5.2. Sequences were concatenated [3]. Nuclear ribosomal gene analyses were performed in Mega6 [18] using maximum likelihood (ML) with Tamura–Nei and GTR+I as the best model with 1000 bootstrap replicates, and maximum parsimony (MP) with 1000 bootstrap replicates was also carried out. These phylogenetic analyses were supported by the Bayesian approach with MrBayes v. 3.1.2 from the CIPRES Science Gateway [19]. Two parallel runs of 5,000,000 generations were done with a sampling frequency of 1000 trees. A burnin tree sample of 10 % was discarded. The presented tree was obtained with ML approach. Tree reconstruction, visualization and editing were done with TreeView v. 1.6.6, FigTree v. 1.1.2 and Mega6.

**Table 2:** Primers and PCR conditions

Gene	PCR primers [references]	PCR condition
*SSU*		
Amplification	NS1 [11], Oli04 [12] NS24 [13]	95 °C 5 min, 35 cycles (95 °C 45 s, 48 °C 40 s, 72 °C 2 min), 72 °C 10 min
Sequencing primers	Oli03 [12], BF83, BF951, BF 1419, BF963, BF1438 [14]	95 °C 1 min, 30 cycles (95 °C 10 s, 50 °C 5 s, 60 °C 4 min)
*ITS*		
Amplification primers	ITS4, ITS5 [11] V9G [15], LS266 [16]	95 °C 5 min, 35 cycles (95 °C 35 s, 48 °C 30 s, 72 °C 1 min), 72 °C 4 min
Sequencing primers	ITS4, ITS5 [11] V9G [15], LS266 [16]	95 °C 1 min, 30 cycles (95 °C 10 s, 50 °C 5 s, 60 °C 4 min)
*LSU*		
Amplification primers	LROR, LR7 [17]	95 °C 5 min, 35 cycles (95 °C 45 s, 48 °C 40 s, 72 °C 2 min), 72 °C 10 min
Sequencing primers	LROR, LR5, LR7 [17]	95 °C 1 min, 30 cycles (95 °C 10 s, 50 °C 5 s, 60 °C 4 min)

## Results

The ITS characters of the three investigated strains deviated significantly from those of known species, both in length and in G+C %: CBS 112.97 had 603 bp, 58.04 G+C %; CBS 100442 had 599 bp, 57.43 G+C %; and CBS 206.96 had 554 bp, 61.55 G+C %. This G+C % of CBS 206.96 is the highest comparing to all *Ochroconis* and *Verruconis* species (Table 3).

**Table 3:** ITS characters of *Ochroconis* and *Verruconis* species

Strain	Species	ITS characters	
		Length (bp)	G+C %
CBS 100442	*Ochroconis bacilliformis* sp. nov.	599	57.43
CBS 206.96	*O. phaeophora* sp. nov.	554	61.55
CBS 112.97	*O. robusta* sp. nov.	603	58.04
CBS 284.64	*O. anellii*	649	51.16
CBS 131816	*O. anomala*	754	58.49
CBS 120700	*O. crassihumicola*	514	56.03
CBS 202.27	*O. constricta*	640	52.19
CBS 475.80	*O. cordanae*	566	56.54
CBS 119644	*O. globalis*	687	57.06
CBS 239.78	*O. gamsii*	678	53.54
CBS 116655	*O. humicola*	674	54.90
CBS 536.69	*O. icarus*	591	58.71
CBS 131815	*O. lascauxensis*	584	59.93
CBS 435.76	*O. longiphora*	651	56.22
CBS 102491	*O. macrozamiae*	668	51.80
CBS 510.71	*O. minima*	580	59.31
CBS 729.95	*O. musae*	643	54.12
CBS 137170	*O. olivacea*	704	57.10
CBS 137173	*O. ramosa*	606	59.74
CBS 135765	*O. sexualis*	592	48.82
CBS 100438	*O. tshawytschae*	707	60.96
CBS 383.81	*O. verrucosa*	699	55.36
CBS 125818	*Verruconis calidifluminalis*	668	51.80
CBS 437.64	*V. gallopava*	669	52.32
CBS 119775	*V. verruculosa*	597	60.47

**Fig. 1:**
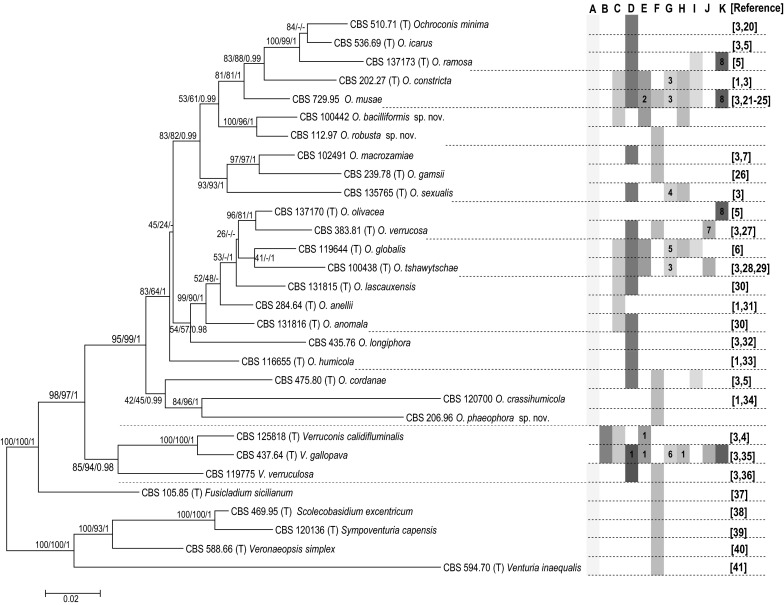
Mega6 maximum likelihood (ML) tree based on a dataset comprising concatenated gene regions of nuSSU, ITS and nuLSU of *Ochroconis*, *Verruconis* and their neighboring genera. The growth profile, associated habitat and pathogenicity information collected from the members of each species have been incorporated into the figure. Numbers at the branches are bootstrap values (%) for Mega6-ML and Mega6-MP, and Bayesian posterior probabilities (PP); ML/MP/PP. Type strains are indicated by (T). *A* mesophilic, *B* thermophilic, *C* oligotrophic, *D* soil saprophytic, *E* water associated, *F* plant associated, *G* animal associated, *H* domestic associated, *I* superficial and cutaneous infections in human, *J* subcutaneous infection in human, *K* systemic infection in human, *1* high temperature, *2* including sea water, *3* cold-blooded animal infection, *4* insect colonization, *5* cold-blooded animal infection and insect colonization, *6* warm-blooded animal infection, *7* unpublished data, *8* isolated from clinical specimens, nonsterile site of deep organ; BAL, sputum, etc

With analyses of partial genes and spacers of the nuclear ribosomal operon, the alignment contained 4103 characters (1656 bp from the SSU, 1040 bp from the ITS, 1407 bp from the LSU) of which 788 were parsimony informative. Applying the algorithms mentioned above, the three investigated strains cluster with *Ochroconis* spp. in a well-supported clade (95 %ML/99 %MP/1PP) but in different branches (Fig. 1). Single-gene analyses of SSU, ITS or LSU revealed that the three strains invariably remained separate from all known *Ochroconis*, *Verruconis* and neighboring species (data not shown). CBS 112.97 and CBS 100442 were strongly supported as sister species (100 %ML/96 %MP/1PP) and found positioned in a cluster containing *O. musae*, *O. constricta*, *O. minima*, *O. ramosa* and *O. icarus* (53 %ML/61 %MP/0.99PP). CBS 206.96 clustered as a sister species of *O. crassihumicola* (84 %ML/96 %MP/1PP) and was paraphyletic to *O. cordanae* (42 %ML/45 %MP/0.99PP). The significant phylogenetic distances and differential morphological characters underlined that the strains represented three hitherto undescribed species members of the genus *Ochroconis*.

## Taxonomy

**Fig. 2:**
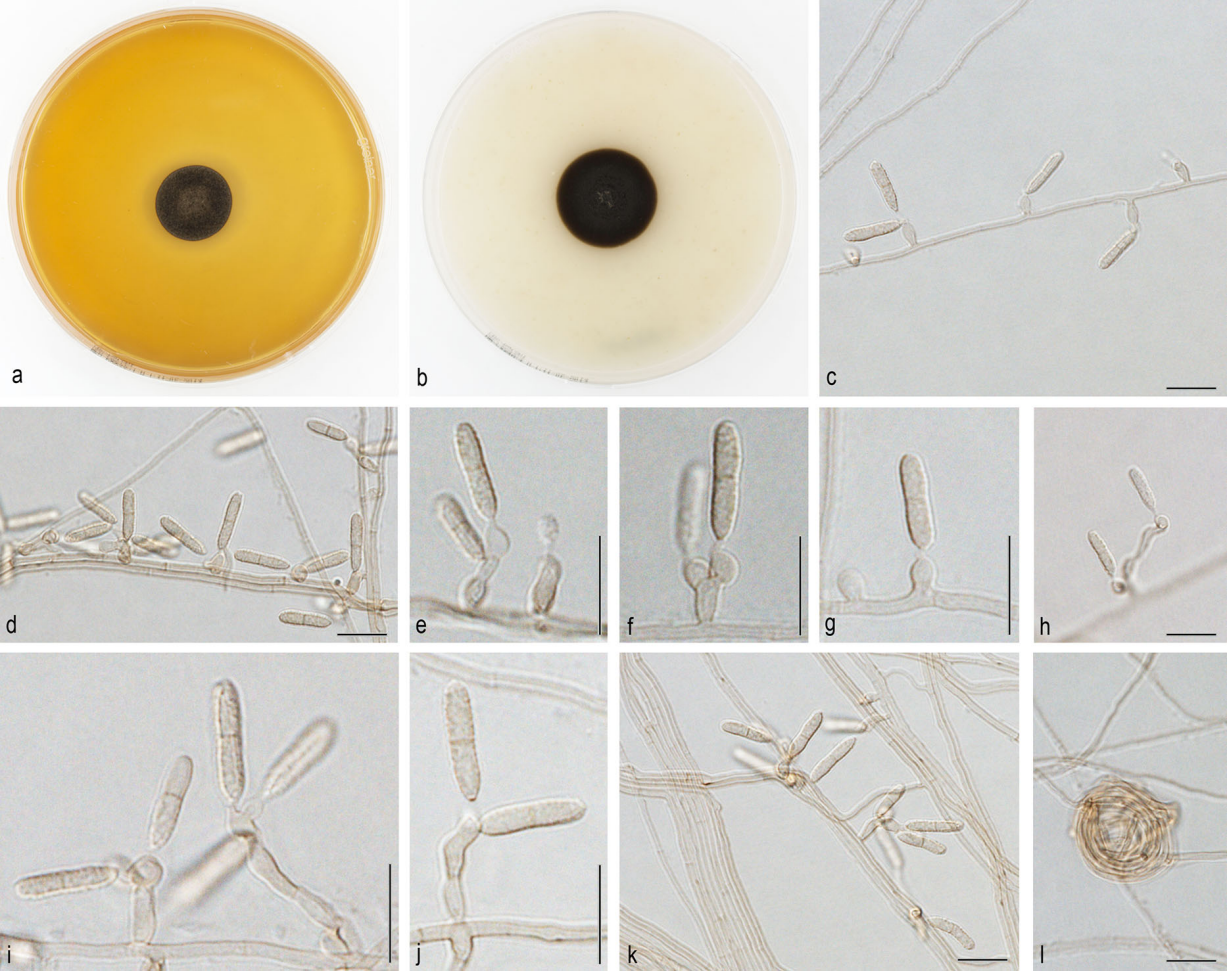
*Ochroconis bacilliformis*, CBS 100442. **a** **b** Colony on MEA and OA after 3 weeks, respectively. **c**–**j**. Conidiophores with conidia. **k**, **l** Conidial apparatus, hyphae, anastomosing hyphae and hyphal coil. *Scale bar* 10 µm

*Ochroconis bacilliformis* Samerpitak, Gerrits van den Ende, Menken & de Hoog, sp. nov.—MB 810875, Fig. 2.

Etymology: named after its conidial shape.

Specimens examined: Germany, Mülheim, from biofilm on stainless steel in drinking water, 1998, collected by E. Göttlich and identified as *Ochroconis constricta*. Holotype CBS H-22032 (dried); ex-type culture CBS 100442 (living) = M 37/2.

Description based on CBS 100442 at 24 °C after 3 weeks in darkness.

On OA, colonies 23–24 mm in diameter, moderately expanding, smooth, dry, flat, dark brown; reverse dark brown. On MEA, colonies 17–18 mm in diameter, flat, velvety to floccose, brown; reverse dark brown. Hyphae subhyaline to pale brown, smooth- and thin-walled, 1–2 μm wide; coiled and anastomosing hyphae usually present. Conidiophores mostly arising laterally from vegetative hyphae, conical, flask-shaped to cylindrical, 3–20 × 2–3 µm, pale brown, smooth-walled, with sympodially proliferating conidiogenous cells each bearing one or a few denticles in the apical region; denticles cylindrical, subhyaline, up to 1 μm long. Conidia cylindrical, rounded at both ends or slightly apiculate at the base, 8.8–12.8 × 2.0–2.4 µm, smooth-walled, pale brown, two-celled, becoming verrucose at maturity. Frills remaining visible on denticle and on conidial base. Cardinal temperatures on MEA: minimum at 4 °C, optimum at 18–24 °C, maximum at 30 °C.

### **Note**

The flask-shaped, rather short conical or cylindrical and pale brown conidiophores of *Ochroconis bacilliformis* are similar to those of *O. constricta* and *O. minima*. Its specific character is the conidial shape which is different to Y- to T-shaped or lobate conidia of *O. minima*, but somewhat similar to that observed in *O. constricta*, but conidia of *O. constricta* are shorter but wider (6–12 × 2.5–4.0 µm) and have constriction at the septum [42].

**Fig. 3:**
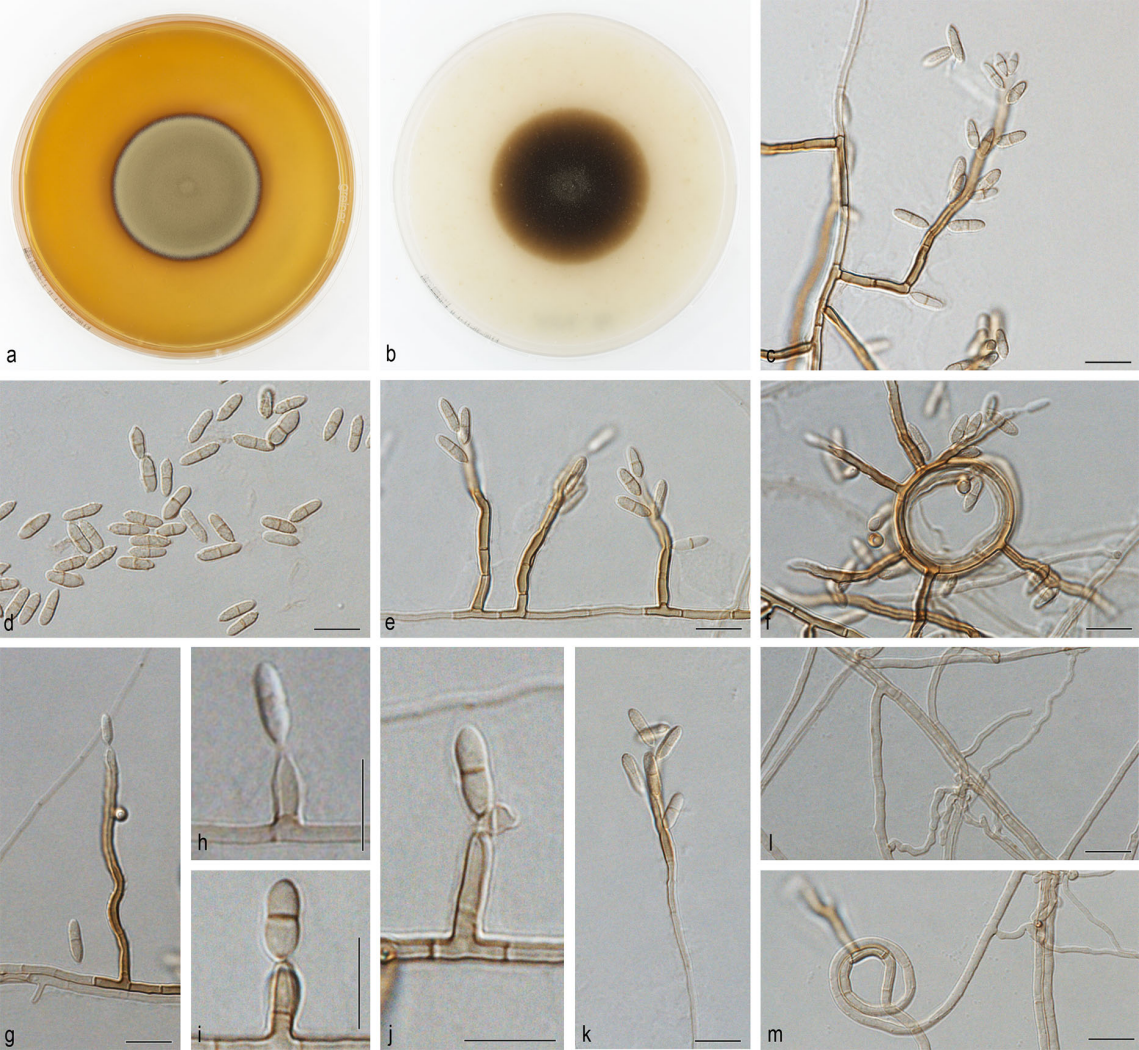
*Ochroconis phaeophora*, CBS 206.96. **a** **b** Colony on MEA and OA after 3 weeks, respectively. **c**–**k**. Conidiophores with conidia. **l**, **m** Anastomosing hyphae and hyphal coil. *Scale bar* 10 µm

*Ochroconis phaeophora* Samerpitak, Gerrits van den Ende, Menken & de Hoog, sp. nov.—MB 810876, Fig. 3.

Etymology: named after its dark brown conidiophores.

Specimens examined: Papua New Guinea, Madang, Balek, from leaf in coastal rain forest, 1995, collected by A. Aptroot and A. van Iperen and identified as *Ochroconis humicola*. Holotype CBS H-22033 (dried); ex-type culture CBS 206.96 (living) = 36599/No. A 165.

Description based on CBS 206.96 at 24 °C after 3 weeks in darkness.

On OA, colonies 40–41 mm in diameter, moderately expanding, smooth, dry, flat, brown to dark brown; reverse dark brown. On MEA, colonies 37–40 mm in diameter, velvety, brown; reverse dark brown with a dark pinkish pigment exuding into the agar. Hyphae subhyaline to pale brown, smooth- to rough- and thick-walled, 1.2–2.8 μm wide; coiled and anastomosing hyphae usually present. Conidiophores mostly arising laterally from vegetative hyphae, erect or flexuous, short- to long-cylindrical with 1–3 (–5) septa, 6–68 × 2.0–2.8 µm, pale brown to dark brown, initially smooth- and thin-walled, rough- and thick-walled at maturity, with sympodially proliferating conidiogenous cells bearing one or more denticles in the apical region; denticles cylindrical, subhyaline, up to 2 μm long. Conidia cylindrical to slightly fusiform, sometimes constricted at the septum, 7.2–11.6 × 2.0–3.6 µm, smooth-walled, pale brown, two-celled, becoming verrucose at maturity. Frills remaining visible on denticle and on conidial base. Cardinal temperatures on MEA: minimum at 4 °C, optimum at 24 °C, maximum at 30 °C.

### **Note**

This species has conidial morphology close to that of *O. crassihumicola* (CBS 120700), but the conidia of the latter species are longer and wider (7.5–13.0 × 4.2–5.5 µm) and mostly rounded at both ends [34].

**Fig. 4:**
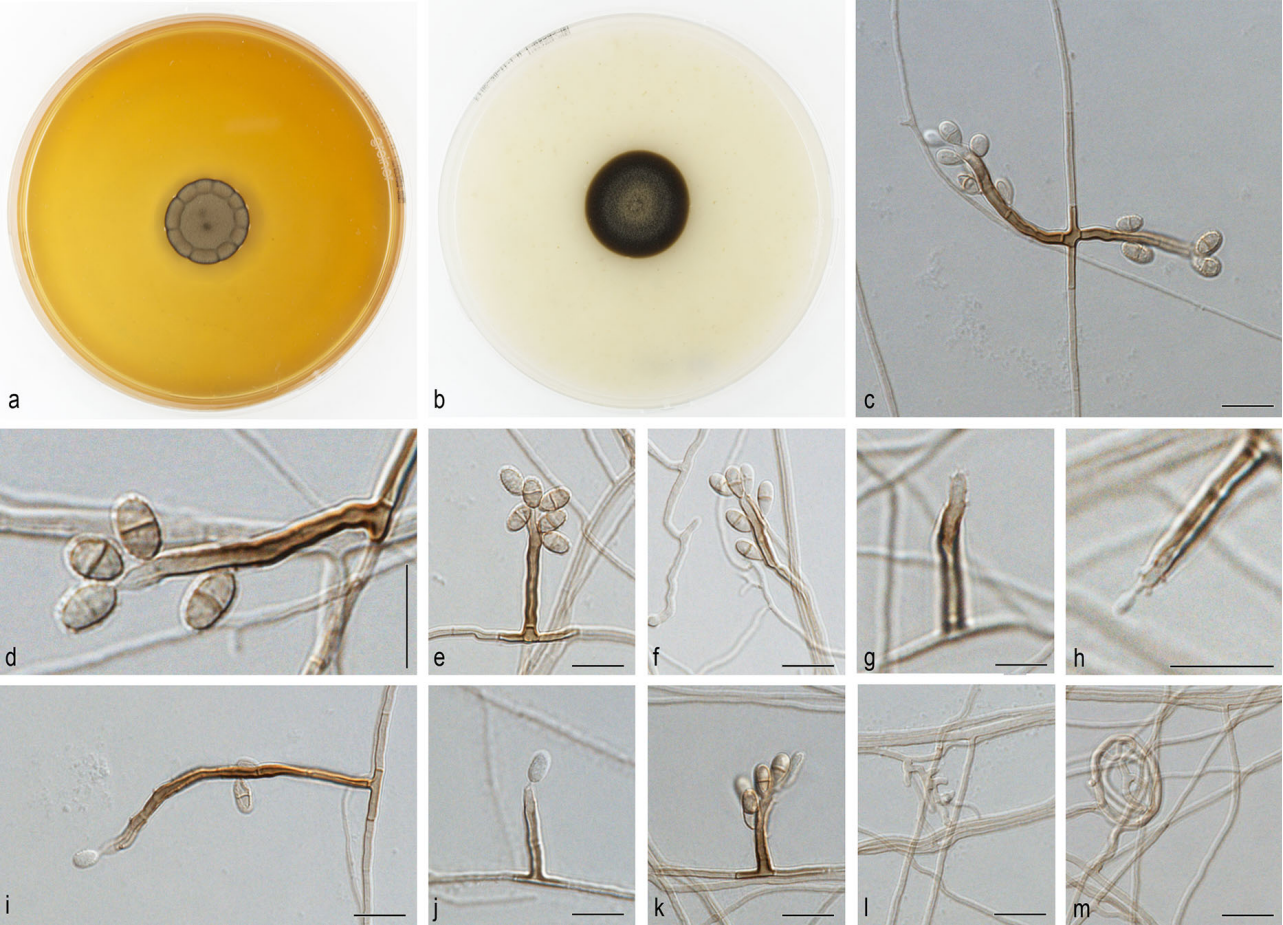
*Ochroconis robusta*, CBS 112.97. **a**, **b** Colony on MEA and OA after 3 weeks, respectively. **c**–**k** Conidiophores with conidia. **l**, **m** Anastomosing hyphae and hyphal coil. *Scale bar* 10 µm

*Ochroconis robusta* Samerpitak, Gerrits van den Ende, Menken & de Hoog, sp. nov.—MB 810874, Fig. 4.

Etymology: named after the well-differentiated conidiophores.

Specimens examined: Spain, from leaf litter of *Ouercus ilex*, 1996, collected by R.F. Castañeda and identified as *Ochroconis humicola*. Holotype CBS H-22031 (dried); ex-type culture CBS 112.97 (living) = INIFAT C96/119.

Description based on CBS 112.97 at 24 °C after 3 weeks in darkness.

On OA, colonies 23–24 mm in diameter, dry, flat, dark brown, velvety at the center, smooth at the margin; reverse dark brown. On MEA, colonies 19–21 mm in diameter, velvety with regular shallow radial fissures at the margin, brown; reverse dark brown. Hyphae subhyaline to pale brown, smooth-walled, 1–2 μm wide; coiled and anastomosing hyphae usually present. Conidiophores arising laterally and terminally from vegetative hyphae, erect or flexuous, cylindrical with 1–3 septa, 10–60 × 1.5–2.0 µm, dark brown, smooth- and thick-walled, with sympodially proliferating conidiogenous cells bearing one or more denticles in the apical region; denticles cylindrical, subhyaline, up to 1 μm long. Conidia ellipsoidal to cylindrical, rounded at both ends or slightly apiculated at base, 4.4–6.8 × 2.4–4.0 µm, two-celled, pale brown, smooth-walled, becoming verrucose at maturity. Frills remaining visible on denticle and on conidial base. Cardinal temperatures on MEA: minimum at 4 °C, optimum at 21–24 °C, maximum at 30 °C.

### **Note**

*Ochroconis robusta* has conidiophores that resemble those of *O. humicola*; in fact, the strain was originally identified as such. However, the shapes of their conidia are different, and *O. humicola* produces longer cylindrical conidia (7–15 × 2.5–4.0 µm) [33], while conidia of *O. robusta* are similar to those observed in *O. cordanae*, *O. globalis* and *O. anomala*. Some subtle morphological characters may help to differentiate such as *O cordanae* has obovoidal to broadly fusiform conidia [3] and *O. anomala* produces short chains of conidia [30].

## Discussion

Ribosomal gene analyses were sufficient to recognize the three strains, CBS 112.97, CBS 206.96 and CBS 100442, as separate, undescribed species showing strong support of their taxonomic positions within the genus *Ochroconis*. Phylogenetic analyses of partial coding genes, actin (*ACT1*), β-tubulin (*BT2*) and translation elongation factor 1-α (*TEF1*) were difficult to apply due to high degrees of variability which interfered with alignment over the entire genus. The large phylogenetic distances between species strongly support their identities as novel taxa in the *Sympoventuriaceae* and suggest that a possible existence of a large number of as yet unrecognized taxa would be waiting to be discovered in unexplored habitats.

With BLAST searches in GenBank, no sequence identical to any of the investigated genes of the new species was encountered. Consequently, although each of the new species is represented by a single strain, their novelty is unambiguous. However, given the morphological variations found upon different culture conditions, identification of these species on the basis of morphology alone remains difficult. Members of the genus have comparable phenotypes despite significant phylogenetic distance between taxa. Nuclear ribosomal sequences of ITS and LSU and even the conserved SSU gene are all usable as diagnostic tools for species identification in *Ochroconis*. It is remarkable that in the *Sympoventuriaceae* (including the genera *Ochroconis*, *Verruconis*, *Veronaeopsis* and *Sympoventuria*), all ribosomal genes, viz. ITS, LSU and the highly conserved SSU, are suitable for identification [3]. This is exceptional in ascomycetous fungi which often share identical ribosomal operons between species, e.g., in *Penicillium* [43] and *Fusarium* [44]. ITS sequences with BLAST searches in GenBank directly led to presumptive identification of *Ochroconis* and *Verruconis* species. In addition, specific characters in length and G+C % among the pathogenic species (Table 3) provide potentiality to ITS sequences to be developed as tool for definite diagnosis in the medical laboratory.

During the last decade, several reports of new species members in the genus *Scolecobasidium*—which was considered as of doubtful identification [3]—have appeared such as *S. chinense* [45], *S. qinghaiense* [45], *S. microsporum* [46], *S. pallescens* [47], *S. tuberculatum* [48] and *S. rostricola* [49]. These species all share similar morphology with existing *Ochroconis* species. Their two-celled conidia may resemble *O. robusta* and *O. phaeophora*, but seem to be different in the details and other phenotypic characters. None of them has conidia which are apiculate at both ends, *Scolecobasidium chinense* has branched conidiophores, *S. microsporum* has conidia with fine spines at the surface, *S. pallescens* has caespitose conidiophores, *S. qinghaiense* has conidia with constricted septa, *S. tuberculatum* has oblong and tuberculate conidia, and *S. rostricola* is a fungicolous hyperparasite. The DNA sequences of these *Scolecobasidium* species are not available in GenBank, and no authentic material was available for study.

Given the limited number of strains per species available, ecological hypotheses are formulated with difficulty. Two species, *O. robusta* (CBS 112.97) and *O. phaeophora* (CBS 206.96), are likely to represent plant-associated saprobes, the former having been isolated from leaf litter from Spain and the latter from the leaf under conditions of a tropical rain forest in Papua New Guinea. *Ochroconis bacilliformis* (CBS 100442) colonized a metal surface submerged in a municipal drinking water network and thus seems to be an oligotrophic saprobe similar to the domestic indoor wet cell colonizers, *O. musae*, *O. constricta*, *O. globalis* [3, 6, 23–25], to the species occurring on cold moist rock, *O. anellii* [31], *O. anomala* and *O. lascauxensis* [30], and also to the waterborne species, *O. tshawytschae* [3]. Waterborne *Ochroconis* species have repeatedly been reported as opportunistic agents of disease in cold-blooded animals that complete at least a part of their life cycle in water [22, 28, 29, 50] and may cause superficial infections in humans with impaired blood circulation [5, 29]. Given the identification problems when using phenotypic characteristics, the species introduced in this paper may be likely to have similar abilities.
